# Immune dysregulation in type 2 diabetes mellitus: Implications for tuberculosis, COVID-19, and HIV/AIDS

**DOI:** 10.1016/j.imj.2025.100211

**Published:** 2025-10-06

**Authors:** Uzair Abbas, Harendra Kumar, Niaz Hussain, Ishfaque Ahmed, Rabeel Nawaz Laghari, Misha Tanveer, Mohammad Hadif, Kashaf Fatima, Muhib Ullah Khalid, Khadija Anwar, Mahtab Khan

**Affiliations:** a Department of Physiology, Dow University of Health Sciences, Karachi 74200, Pakistan; b Dow International Medical College, Dow University of Health Sciences, Karachi 74200, Pakistan; c Bilawal Medical College, Liaquat University of Medical and Health Sciences, Jamshoro 76080, Pakistan; d Sindh Infectious Diseases Hospital and Research Center, Dow University of Health Sciences, Karachi 75100, Pakistan; e Department of Medicine, Indus Medical College Hospital, Tando Muhammad Khan 70220, Pakistan

**Keywords:** Diabetes mellitus, Immune modulation, Dysregulation, Tuberculosis, COVID-19, HIV/AIDS, Vaccine

## Abstract

Diabetes mellitus (DM) is a complex and multifactorial disorder associated with elevated blood sugar levels, poor insulin sensitivity, and inadequate insulin production. It has a major impact on the immune system, making a person more susceptible to and influenced by a variety of infectious illnesses. This narrative review summarizes the relationship between chronic inflammation and high glucose levels in DM, on susceptibility and outcomes in endemic infectious diseases. We focused on impact of DM on disease progression, and treatment response in these infections. Literature was identified through searches of PubMed, Scopus, and Google Scholar, focusing on epidemiologic, clinical, and mechanistic studies. The evidences suggest that immune modulation in DM has profound inverse relations with the outcome of infectious diseases including tuberculosis, COVID-19, and HIV/AIDS. DM increases the risk of developing severe forms of infectious diseases due to downregulation of the immune system which is associated with glycemic control. There is a need to understand the relationship between DM and immunological control for developing methods to reduce these risks and improve outcomes for the affected population.

## Highlights

• In this review, we have summarized how chronic hyperglycemia impacts immunity against pathogens.
• This review summarizes the immune modulation in diabetes mellitus including immunogenetics of diabetic population.
• This review shows how immune modulation in diabetes mellitus impacts the susceptibility of getting infections like tuberculosis, COVID-19 and HIV and the outcome of these infections.
• This will help in understanding the phenotype of infections in diabetic population and formulate a different patient care specially in high endemic countries.

## 1 Introduction

Diabetes mellitus (DM) is a chronic metabolic disorder with its global burden of around half a billion in 2017 with an expected rise of 7,079 cases per 100,000 individuals by the year 2030.^1^ DM is characterized as persistently elevated blood sugar levels caused by issues with insulin production, effectiveness, or both. Although the fundamental impacts of DM are well understood which affects the cardiovascular system, or causes neuropathy, nephropathy, and retinopathy, one crucial but often overlooked aspect of DM is its significant influence on the immune system.^2^^,^^3^ Diabetic patients' immune systems are significantly altered as a result of persistent hyperglycemia and associated metabolic dysregulations.^4^ This immunological regulation weakens the body's natural defensive systems, making diabetic population more susceptible to a variety of infectious diseases.^2^

DM and infectious diseases have a complicated and biased interaction.^5^ While infections may impair glycemic management and cause acute issues in diabetic individuals, on other hand immunological dysfunction associated with DM makes them more susceptible to infections.^6^ DM patients are more prone to getting infections such as tuberculosis (TB), HIV, dengue, urinary tract infections, respiratory tract infections, and even more severe versions of common diseases like influenza and pneumonia.^7^ In general, these infections cause more severe symptoms, longer illness courses, and a greater risk of complications than non-diabetic persons (Table 1). For example, TB in diabetics is linked with more severe lung damage, a greater bacterial load, and a delayed therapeutic response, complicating therapy and increasing the chance of treatment failure.^6^^,^^8^

**Table 1:** Mechanisms of immune modulation in DM.

Immune modulation pathway	Mechanism	Effect on immune function	Example of infectious disease impact	References
Hyperglycemia-induced dysfunction	High blood glucose impairs neutrophil function	Reduced phagocytosis, impaired killing of pathogens	Bacterial skin infections, pneumonia	^[^^9^^,^^10^^]^
Altered cytokine production	Pro-inflammatory cytokines (IL-6, TNF-α) dominate	Chronic inflammation, impaired immune resolution	Increased severity and frequency of viral infections (e.g., influenza, COVID-19)	^[^^11^^,^^12^^]^
Increased inflammatory response	Chronic inflammation from persistent hyperglycemia	Dysregulated immune response, tissue damage	Increased mortality from sepsis	^[^^9^^,^^11^^]^
Decreased T-cell function	High glucose impairs T-cell activation and response	Reduced adaptive immunity	Poor response to viral infections, tuberculosis	^[^^9^^,^^10^^]^

*Abbreviations*: IL-6,interleukin 6; TNF-α,tumor necrosis factor-α.

The immunological dysregulation in both type 1 and type 2 DM (T1DM and T2DM) causes a decrease in functionality of several immune cells, including neutrophils, lymphocytes and importantly—the macrophages, which are critical for the body's defense against infections.^2^^,^^4^ Both types of DM dysregulate the immune system through different pathways. Ultimately, hyperglycemia disrupts the normal action of these cells, reducing chemotaxis, phagocytosis, and microbial death, hence reducing the body's capacity to successfully combat with infections.^5^ Furthermore, the chronic inflammatory state caused by DM raises the chance of infection and leads to harmful outcomes.^4^ This dysregulated immune response not only raises the incidence of infections, but also influences the course and severity of these illnesses, affecting clinical outcomes and increasing death rates in diabetics.^5^

Understanding link between DM and immune response in them is critical, given the rising worldwide incidence of DM and the increasing burden of infectious illnesses.^5^ Understanding how DM impacts immune function and affects the susceptibility and consequences of infectious illnesses might help healthcare practitioners develop more effective techniques for early identification, infection treatment, and prevention in diabetic populations.^6^ T2DM, being more prevalent, this narrative review aims to emphasize the complex relationship between T2DM and immunological control, with a focus on how these alterations influence susceptibility to chronic infections and affect the clinical outcomes. This review will emphasize the importance of personalized methods to minimize infections in diabetic patients, ultimately improving patient care and outcomes in this high-risk population, based on an exhaustive examination of existing data. Here we discuss how the immune system is dysregulated in DM and how it impacts the outcome of infectious diseases.

## 2 Immunity in DM

### 2.1 Immune dysregulation in T1DM

T1DM is an autoimmune disease in which loss of β-cells and absolute insulin deficiency occurs on a background of systemic immune imbalance. While the autoimmunity is antigen-specific (islet antigens), the downstream immune phenotype is global: altered innate cell function, skewed T-cell responses, checkpoint/tolerance defects, and cytokine imbalance.^13^ Superimposed hyperglycemia and glycation further impair host defense. Together, these changes raise infection risk and can blunt vaccine and pathogen-specific responses.^14^

T1DM leads to dysregulated myeloid cells including macrophages and dendritic cells. Dendritic cells show altered maturation and increase co-stimulation, facilitating priming of effector T cells but not improving antimicrobial killing.^15^ Studies show quantitative/functional abnormalities of neutrophils and natural killer (NK) cells potentially diminishing early antiviral control.^16^

T1DM also affects adaptive immune response through T-helper (Th) 1/Th17 polarization leading inefficient antimicrobial responses and further on pathogen exposure leads to T-cell dysfunction or exhaustion.^17^ Moreover, both the frequency and suppressive function of regulatory T cells (Tregs) are impaired. These cells normally maintain immune tolerance and control excessive inflammation through the secretion of immunoregulatory cytokines such as interleukin (IL)-10 and tumor growth factor (TGF)-β. However, in T1DM, Tregs exhibit reduced suppressive capacity and altered cytokine production.^18^ This dysregulation lowers the threshold for inflammatory responses during infection, leading to exaggerated immune activation, while simultaneously impairing the ability to restore homeostasis and resolve inflammation. Such defects contribute to ineffective pathogen clearance and increased tissue damage.^19^

In individuals at risk of developing T1DM, type I interferons (IFN-α and IFN-β) are often elevated in the blood and in pancreatic islets. The systemic immune environment in T1DM is biased toward inflammation.^20^ Elevated levels of IL-1β, tumor necrosis factor (TNF)-α, and IL-6 drive ongoing immune activation and tissue stress, while IL-10, a key anti-inflammatory cytokine, is relatively reduced.

Type I interferons which are chronically elevated in T1DM, can suppress protective IL-12 and IFN-γ responses that are crucial for macrophage activation and *Mycobacterium tuberculosis* (Mtb) control.^21^ Hence the pro-inflammatory cytokine milieu does not translate into effective granuloma formation; instead, macrophage function is impaired leading to poor containment of Mtb. The net result is weaker granulomas, higher risk of reactivation TB, and more severe disease progression compared to non-diabetic individuals.^22^

During respiratory viral infections like SARS-CoV-2, this inflammatory bias can amplify lung injury and worsen clinical outcomes, without necessarily enhancing viral clearance.^23^ The systemic inflammatory predisposes patients to a hyperinflammatory response (cytokine storm) once infection is established. Immune cell defects reduce viral clearance and increased risk of severe pneumonia, prolonged recovery, and higher mortality in T1DM COVID-19 patients compared with the general population.^24^

In T1DM, the pro-inflammatory cytokine profile accelerates chronic immune activation, which synergizes with HIV-driven inflammation. Reduced IL-10 and defective Tregs leads to faster CD4⁺ T-cell decline. Type I interferons, although antiviral, become maladaptive in chronic HIV, as their persistent signaling promotes immune exhaustion and impaired CD8⁺ cytotoxicity.^25^ Clinically, this leads to poorer immune recovery under antiretroviral therapy (ART), higher risk of opportunistic infections, and faster progression to AIDS in T1DM patients co-infected with HIV.

### 2.2 Immune dysregulation in T2DM

In T2DM, obesity and metabolic stress induce chronic low-grade inflammation. This inflammation is characterized by infiltration of macrophages into adipose tissue, increased proinflammatory cytokines (TNF-α, IL-6, IL-1), and activation of innate immune pathways. These cytokines impair insulin signaling, leading to insulin resistance. Insulin resistance then causes hyperglycemia especially when pancreatic β-cell compensation fails.^26^^,^^27^

In the innate immune system, hyperglycemia may set off the nucleotide-binding domain, leucine-rich–containing family, pyrin domain–containing 3 (NLRP3) inflammasome to activate, therefore releasing more IL-1 and producing more inflammation. Moreover, generated in DM are reactive oxygen species (ROS) and advanced glycation end products, which activate nuclear factor-kappa β (NF-κβ) pathways and thus aggravate the inflammatory response.^4^^,^^6^ This inflammatory environment reduces neutrophil function, including phagocytic activity and reduced chemotaxis, therefore compromising the body's first line of protection against infections.^8^

A faulty immune response results from dysregulation of cytokines including IFN-γ and altered expression of immune-regulating genes like suppressor of cytokine signaling (SOCS) 1 and 3 in the adaptive immune system. Increasing in DM, SOCS proteins negatively control cytokine signaling and prevent effective T cell and other immune cell activation.^4^^,^^8^ This instability affects the physiology of Th cells, mainly Th1 and Th17 subsets, which are very important for generating effective immune responses to infections.^28^ The impact of DM on immune cells and its impact on infection susceptibility are summarized in Table 2.

**Table 2:** Immune system alterations in diabetes mellitus.

Immune cells	Normal function	Altered function in diabetes	Impact on infection susceptibility	References
Neutrophils	First responders in infection	Impaired chemotaxis, reduced phagocytosis	Increased susceptibility to bacterial infections	^[^^10^^,^^30^^,^^31^^]^
Macrophages	Engulf pathogens, present antigens to T cells	Delayed and reduced response	Delayed clearance of infections	^[^^8^^,^^32^^,^^33^^]^
T cells	Coordinate immune response, cytotoxic activity	Reduced activation and proliferation	Increased risk of viral infections	^[^^8^^,^^34^^,^^35^^]^
Cytokine profile	Regulates immune responses (balance of pro- and anti-inflammatory)	Dysregulated cytokine release, pro-inflammatory dominance	Persistent inflammation, worsened infection outcomes	^[^^30^^,^^33^^,^^34^^]^

**Fig. 1:**
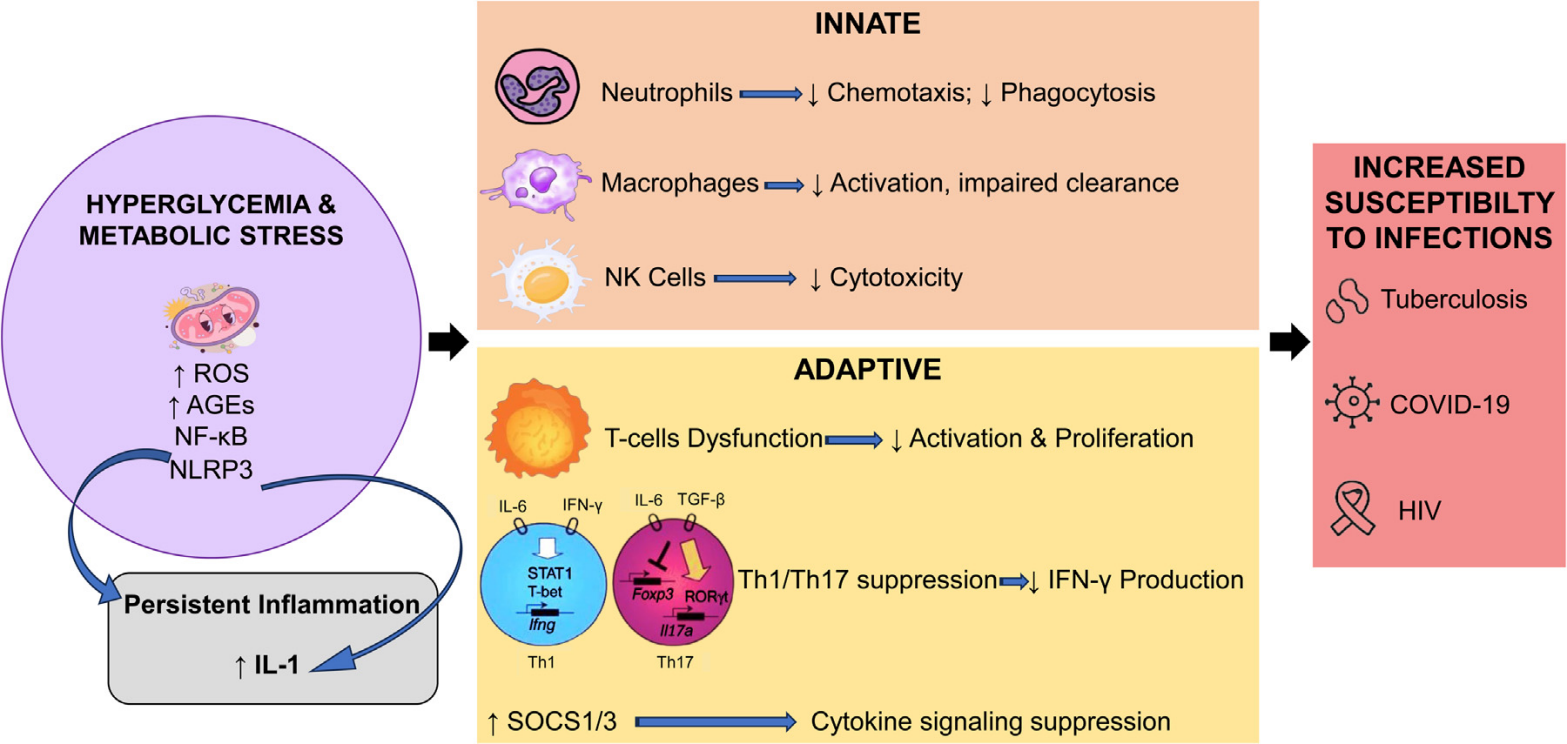
The impact of DM on innate and adaptive immune responses and susceptibility to infectious diseases. *Abbreviations*: ROS, reactive oxygen species; AGE, advanced glycation end product; NF-κB, nuclear factor kappa-light-chain-enhancer of activated B cells; NLRP3, nucleotide-binding domain, leucine-rich–containing family, pyrin domain–containing 3; IL-1, interleukin-1; IFN, interferons; SOCS, suppressor of cytokine signaling.

Moreover, the chronic inflammatory state in DM causes immune cells—especially T cells and NK cells—to be decreased, therefore lowering their potential to respond to infections.^29^ Apart from reduced immune cell activity, the altered balance of pro- and anti-inflammatory cytokines creates a disorganized immunological milieu in DM patients that increases their vulnerability to infections and reduces the possibility of developing strong immune responses. Customizing treatments for diabetic patients to control immunological reactions and improve clinical results requires an understanding of these pathways.^26^ The impact of DM on innate and adaptive immune responses and susceptibility to infectious diseases is summarized in Fig. 1.

### 2.3 Immunogenetics of DM

The genetic and immunogenetic alterations in T2DM not only predispose individuals to metabolic dysregulation but also exacerbate susceptibility to endemic infectious diseases including TB, pneumonia, COVID-19, and HIV. These conditions share overlapping immunological pathways, where genetic predisposition in T2DM patients contributes to impaired immune responses, chronic inflammation, and vulnerability to infectious agents.

Genome-wide association studies have reported multiple loci associated with T2DM risk. Those genes regulate insulin secretion, glucose metabolism, and pancreatic β-cell function. For example, transcription factor 7 like 2 *(TCF7L2)* is the most potent T2DM influencing β-cell function. Variation in *TCF7L2* contributes to hyperglycemia, which in turn impairs immune cell function, rendering individuals more susceptible to infections by reducing the metabolic state required effective immunologic responses.^36^^,^^37^ The fat mass and obesity-associated *(FTO)* variant rs9939609 has been linked to increased obesity and T2DM risk in Asian Indians, with its effect on T2DM partly mediated through BMI but highlighting unique adiposity patterns in this population.^38^ Obesity, associated with *FTO* is a pro-inflammatory state that aggravates immune dysfunction, leading to a reduced response to pathogens. Epigenetic regulation through DNA methylation of the CpG9 site of the *FTO* gene is also positively associated with T2DM.^39^

Peroxisome proliferator-activated receptor gamma *(PPARG)* is a regulator of lipid metabolism, and insulin functionality and has been implicated in T2DM susceptibility.^40^^,^^41^ Polymorphism in *PPARG* can cause altered lipid metabolism and insulin resistance, leading to a chronic inflammatory environment that decreases immune cell function and increased susceptibility to infections such as pneumonia. Solute carrier family 30-member 8 *(SLC30A8)* gene which encodes for a zinc transporter is highly expressed in the islets of Langerhans. The *SLC30A8* gene polymorphism rs13266634, was associated with lowered beta cell function and an increased risk in diabetes.^42^ Higher DNA methylation levels in the *SLC30A8* gene leads to decreased gene expression, affecting β-cell function contributing to hyperglycemia, which impairs immune function.^42^

Polymorphisms in genes encoding pro-inflammatory cytokines, such as TNF-α, IL-6, and IL-1β which have specific physiological role in glucose metabolism^43^^,^^44^ are associated with chronic low-grade inflammation in T2DM. These changes can amplify inflammatory responses during infections, leading to “cytokine storms” which overwhelm the immune system causing tissue damage, leading to poor outcomes in infectious diseases like TB^45^ and COVID-19.^46^

Genetic variants in toll-like receptor 4^47^^,^^48^ and other pattern recognition receptors (PRRs)^49^ have been linked to altered innate immune responses in T2DM. These mutations can weaken pathogen recognition and clearance, increasing susceptibility to infections like pneumonia and TB, by interfering with the initial detection and signaling pathways essential for effective defenses.

T2DM is associated with a threefold increased risk of TB in low- and middle-income countries due to impaired macrophage and T-cell function.^50^ Genetic susceptibility in T2DM patients, including variants in IFN-γ signaling pathways^51^ and nucleotide-binding oligomerization domain (NOD1 and NOD2) which are PRRs and capable of sensing Mtb,^52^ disrupts protection mechanism against TB. Impaired IFN-γ signaling reduces macrophage activation and regulation of intracellular bacterial growth. NOD mutations can lead to inadequate recognition of mycobacterial components, weakening the hosts' defense against TB.

A study found a substantial metabolic difference between patients with comorbid pneumonia and T2DM and healthy controls or T2DM without pneumonia. The gene set enrichment analysis revealed that significant alterations associated with IL-6 signaling, fatty acid metabolism and oxidative stress, were upregulated in T2DM patients with pneumonia.^53^ This dysregulation, paired with hereditary variables, results in poor neutrophil chemotaxis and phagocytosis, increasing susceptibility *Streptococcus pneumoniae* and other respiratory pathogens by reducing the rapid and effective clearance of bacteria from the lungs.^54^

In COVID-19, the angiotensin converting enzyme 2 *(ACE2)* gene encodes the receptor for SARS-CoV-2 essential for adhesion and uptake of virus into host before replication begins.^55^ In T2DM patients, hyperglycemia and chronic inflammation upregulate *ACE2* expression, facilitating viral entry and replication.^56^ Genetic variations that influence *ACE2* expression, along with metabolic milieu of DM, increases viral infectivity contributing to severe disease course. Furthermore, patients with IL-6 and IL-17A single nucleotide polymorphisms are more prone to severe COVID-19 infection.^57^^,^^58^ T2DM also increases the expression of furin, an enzyme critical for SARS-CoV-2 spike protein cleavage, enhancing viral infectivity and pathogenesis.^59^

Chronic inflammation and persistent immune activation in T2DM can also accelerate HIV progression. Genetic variants in CXCR4,^60^ a receptor for HIV entry, may influence viral replication dynamics in T2DM patients. Pro-inflammatory gene polymorphisms exacerbate immune dysfunction, leading to a faster decline in CD4^+^ T-cell counts and impaired viral suppression, particularly in patients on ART.^61^ These genetic variables combined with diabetes foster an environment that facilitates efficient replication of HIV and greater immunological damage, accelerating disease progression.

## 3 DM and TB

### 3.1 Host immune response to Mtb

Mtb may trigger both adaptive and innate immune responses in humans with or without priming of T cells, which can result in the eradication of the bacteria or the establishment of an infection.^62^ Infection risk, incidence, disease development and its severity depends on environmental factors, co-morbidities, and population demographics.^63^ For a successful infection, Mtb must cross the physical barrier of the airways to reach the lungs where it encounters alveolar macrophages, dendritic cells which activates and recruit adaptive immune cells to restrict bacteria.^64^

The important component of immune response against Mtb is IFN-γ. IFN-γ mediated activation of autophagy leads to phagosome maturation which promotes killing of Mtb due to enhanced acidification.^65^ NK cells identify and kill the infected macrophages. They also further stimulate the production of IFN-γ and other cytokines to increase recruitment of CD8^+^ and NK T cells which ultimately leads to granuloma.^66^ Alveolar macrophages present Mtb antigens to CD4^+^ T lymphocytes via major histocompatibility complex class II molecules resulting in secretion of cytokines such TNF-α and IFN-γ which further activate the innate immune responses, as well as the proliferation of T cells and other lymphocyte activity.^1^^,^^3^ Infection severity and outcome is greatly influenced by morphology of granulomas.

### 3.2 Prevalence of TB in DM and vice versa

The substantially greater prevalence of TB among DM than in the general population highlights a major public health concern. Studies have repeatedly demonstrated that those with DM are 2–3 times more likely than those without the illness to get TB.^67^ The immunosuppressive effects of persistent hyperglycemia, which lowers the body's capacity to mount an effective defense against Mtb, the bacteria that causes TB, might explain this increased risk. Furthermore, the link between DM and TB is complicated and bidirectional.^68^ DM weakens the immune system, increasing susceptibility to TB.^69^ Conversely, TB itself may have a detrimental impact on DM therapy, jeopardizing glucose regulation.^70^ Active tuberculosis stress and inflammation may produce insulin resistance and hyperglycemia, resulting in DM in patients who were previously untreated or pre-diabetic. In extreme situations, TB may aggravate existing DM, complicating treatment efforts and resulting in poorer health outcomes.^51^ The two-way interaction between DM and TB highlights the need for coordinating medical treatments.^71^ Two critical measures in lowering the combined dangers posed by these coexisting disorders are early TB screening in diabetics and cautious blood glucose level control in those with TB. Addressing the confluence of TB and DM is important for improving patient outcomes and halting the spread of TB, especially in places where both illnesses are prevalent.^69^^,^^72^

### 3.3 Impact of hyperglycemia on TB control

Hyperglycemia, a hallmark of DM, makes adequate TB control and treatment difficult.^70^ Elevated blood glucose levels have been proven to damage the immune system, namely by impairing the activity of macrophages and other immune cells that are critical in developing an effective response against Mtb.^51^ Longer illness duration and a greater risk of complications result from the body's more difficult control and clearance of TB infections, which is enabled by this immunosuppressive impact.^71^ Hyperglycemia has an indirect influence on TB therapy, in addition to its direct impact on immunity. The pharmacokinetics and pharmacodynamics of anti-TB medicines may interact with hyperglycemia, complicating the treatment procedure. People with high blood sugar levels, for example, may affect the metabolism and absorption of essential anti-TB medications such as rifampicin and isoniazid, this could lead to subtherapeutic drug levels.^73^^,^^74^ This not only affects treatment effectiveness but also raises the risk of drug resistance, a serious public health concern in TB therapy.^75^

Furthermore, hyperglycemia may exacerbate the inflammatory response associated with TB, exacerbating the sickness symptoms and complicating the clinical course.^76^ Those with poorly managed DM are more likely to have undesirable outcomes such as delayed sputum conversion, increased infectiousness, and higher death rates.^77^ TB and hyperglycemia form a vicious cycle in which one condition exacerbates the other, making it difficult to achieve optimal treatment results.^67^^,^^77^ Aside from these issues, hyperglycemia may influence the TB diagnosis.

**Fig. 2:**
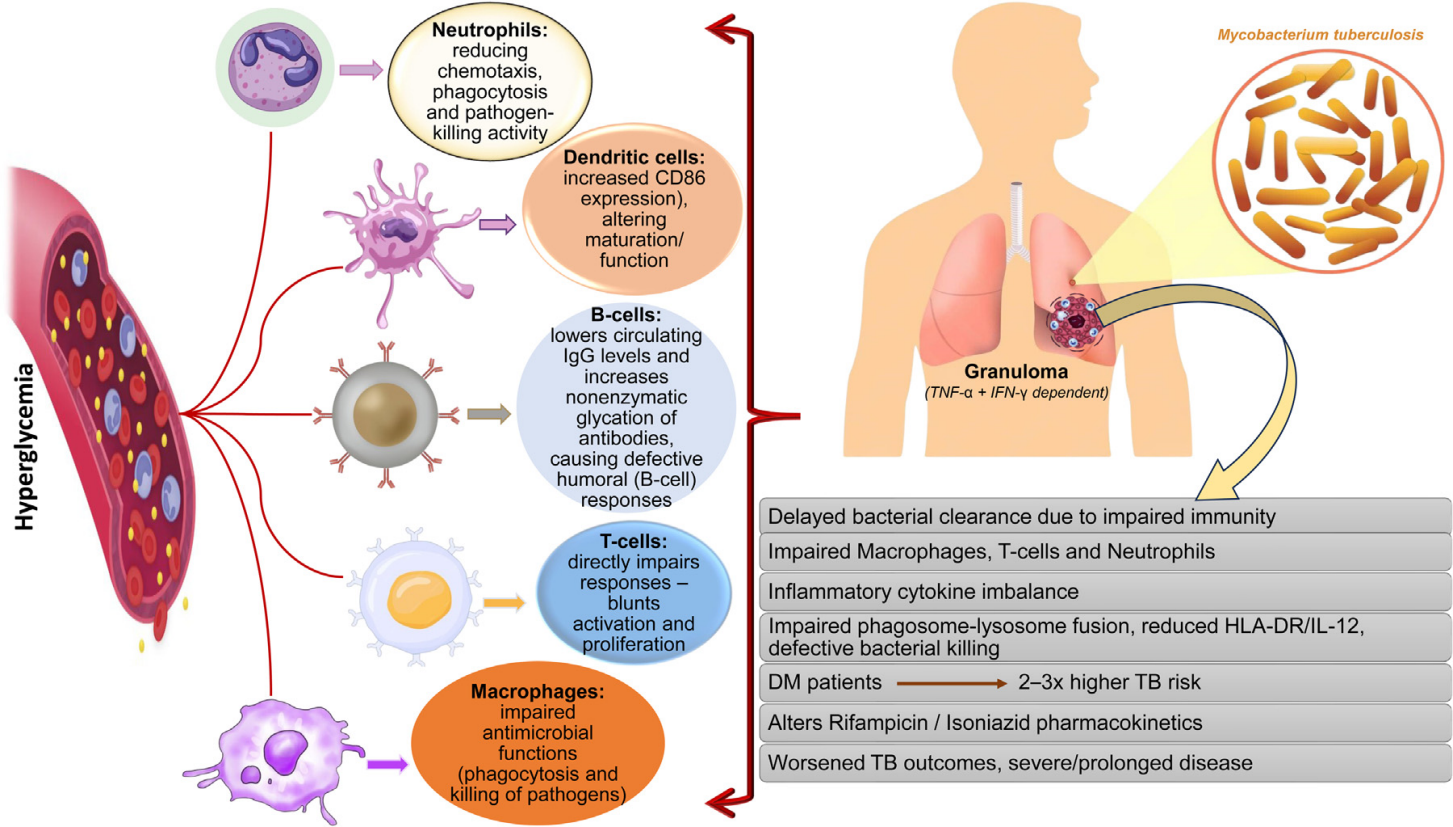
Immune modulation in DM and impact on outcome of TB. *Abbreviations*: DM, diabetes mellitus; TB, tuberculosis; HLA-DR, human leukocyte antigen-DR isotype; IL, interleukin.

DM and TB symptoms often overlap, which might cause delays in diagnosing and treating TB in DM. This delay exacerbates efforts to eliminate TB because untreated or improperly treated TB patients continue to infect others in the community.^78^ Given the many problems, treating hyperglycemia in TB patients is critical for optimal disease management. This necessitates not only effective glycemic management for diabetics, but also consideration of the potential effects of high blood glucose levels when planning and executing TB treatment options.^79^ Incorporating DM management into TB care programs, particularly in places where both illnesses are prevalent, may significantly improve treatment results and lower the cost of TB to public health systems.^80^ The summary of impact of hyperglycemia on immune response against TB and its outcomes is shown in Fig. 2.

### 3.4 Impact of DM on TB treatment

DM has a significant impact on TB therapy, causing a wide range of complications that may have a negative impact on patient outcomes.^81^ The coexistence of these two chronic conditions creates a difficult situation for both healthcare practitioners and patients, as the management of one disease may have a direct effect on the treatment and progression of another.^82^

One of the most important difficulties in treating TB in people with DM is the risk of drug-drug interactions between anti-TB therapies and diabetic medications. For example, rifampicin, a key TB therapy, is known to activate hepatic enzymes, which may accelerate the metabolism of several oral hypoglycemic medications, resulting in poor blood glucose control.^74^ This combination may require changes in DM medication dosages, which can be difficult to manage, especially in resource-limited conditions.^26^ Furthermore, the pharmacokinetics of anti-TB medications may be altered in diabetics, potentially leading to decreased treatment efficacy. Such interactions may lead to inadequate treatment, prolonged infection, and an increased risk of developing drug-resistant TB strains, which are more difficult and costly to treat.^46^

Furthermore, the mutual relationship between TB and DM exacerbates the severity of both diseases, complicating treatment. DM is associated with a weakened immune response, which may impair the body's ability to fight TB infections.^83^ This often results in more severe and broad disease symptoms, such as higher bacterial loads, more extensive lung damage, and a higher risk of extrapulmonary TB.^84^ As a result, diabetics may need longer or more intensive TB treatment regimens, which may be difficult to stick to and increase the risk of serious drug reactions.^72^

The complexities of managing DM and TB also provide significant challenges to patient adherence to treatment regimens.^67^^,^^70^ The need to take many medications, monitor blood glucose levels, and adjust dietary and lifestyle factors may be stressful for patients, particularly in locations where access to healthcare services is limited. Poor adherence to TB or diabetic treatment regimens may result in treatment failure, relapse, or the development of drug resistance, all of which contribute to poor health outcomes.^81^^,^^82^

Moreover, DM has been linked to delayed sputum conversion and a worse response to TB medication, thereby prolonging infectiousness and increasing the risk of transmission within the community.^45^ This delay in treatment response also increases the risk of TB recurrence after medication finishes, necessitating more surveillance and, most likely, longer follow-up care for diabetic patients.^85^

Given these constraints, treating TB in diabetic patients requires a thorough and integrated approach that addresses both conditions simultaneously.^86^ To boost treatment regimen adherence, healthcare practitioners must carefully examine the potential interactions between TB and DM medicines, optimize glycemic control, and provide patient education and support. Furthermore, more frequent monitoring of treatment responses and medication changes may be required to ensure the best possible outcomes for those with comorbid TB and DM.^86^

## 4 DM and COVID-19

### 4.1 Host immune response against SARS-CoV-2

COVID-19 severity varies in different sets of population from not showing any symptoms to severe-critical disease. It mainly impacts the lungs but may also affect other organs.^87^ The virus enters the target host through ACE2 receptors present in several human cells, especially in lung epithelium, kidney, heart, gastrointestinal track and oral mucosal epithelium.^88^

Immune cells initiate signaling cascades after recognizing viral single-stranded RNA or the double-stranded RNA of replicating virus in the cells.^89^ This leads to initiation of type I and III IFN response along with secretion of other chemokines and cytokines that induce further inflammation and help mounting immune response against virus.^90^ However, the virus has ability to disrupt the IFN responses which lead to decreased secretions of these cytokines and cause severe COVID-19.^91^

Elevated lactate dehydrogenase, C-reactive protein, IL-6, d-dimer, white blood cell count, and raised platelet count are important clinical markers of COVID-19 deterioration and ARDS.^92^ Compared to the healthy controls or asymptomatic COVID-19 patients, ICU-admitted patients show increased concentrations of granulocyte colony-stimulating factor (G-CSF), chemokine (C-X-C Motif) ligand 10 (CXCL10), C—C motif chemokine ligand (CCL) 2, CCL3, and TNF which are hallmarks of the “cytokine storm” associated with severe COVID-19.^93^

### 4.2 Prevalence of DM and COVID-19 comorbidity

DM has become a more common comorbidity among COVID-19 patients, with studies indicating that 20%–30% of hospitalized patients had DM.^46^ This frequency underlines the basic relationship between these two disease disorders. DM may significantly impair the immune system's response to SARS-CoV-2, the virus that causes COVID-19.^83^ Hyperglycemia, a feature of DM, may impair immune cell activity, lowering the production and efficacy of key cytokines and antibodies required to combat infections. Aside from greater vulnerability to COVID-19, a weakened immune response typically leads to more severe illness symptoms.^84^

The effect of hyperglycemia on COVID-19 therapy is complicated. Controlling blood glucose levels becomes more difficult when there is stress-induced hyperglycemia and potential interactions between diabetic medications and COVID-19 treatment.^94^ DM has the potential to worsen inflammation and increase thromboembolic events, which complicates COVID-19 therapy and control.

### 4.3 Impact of DM on outcome of COVID-19

In COVID-19, managing the infection and limiting severe disease development are dependent on an early and robust immune response. Early activation of type I and III IFNs, as well as other pro-inflammatory cytokines, reduces viral replication and aids in virus clearance in people with a sound immune system.^4^^,^^8^ This response includes the rapid recruitment and activation of macrophages and NK cells, which are critical for regulating viral multiplication and hence reducing tissue damage.^28^

However, in those with DM, the immune system is often impaired, increasing their risk of severe COVID-19. DM is defined by consistent low-grade inflammation and immunological dysregulation, which results in a less effective initial response to SARS-CoV-2 infection.^34^ Increased pro-inflammatory cytokines, such as TNF-α and IL-6, contribute to a hyperinflammatory condition known as a “cytokine storm”, resulting in an insufficient response. Early in an infection, the chronic inflammatory environment in DM patients inhibits the antiviral immune response, making it more difficult to halt viral replication.^95^

Furthermore, dysregulation of innate immune cells, particularly macrophages and neutrophils, lowers the ability to mount an efficient immunological response.^96^ Delayed or insufficient activation of IFNs and other antiviral cytokines may result in uncontrolled viral growth, significant lung damage, and the emergence of acute respiratory syndrome, a hallmark of severe COVID-19.^97^ Furthermore, the hyperglycemic environment in DM promotes the production of ACE2, the receptor that SARS-CoV-2 employs to enter host cells, increasing the risk of severe infection.^98^

According to a clinical trial, diabetics are much more likely to have severe COVID-19 symptoms such as death, ICU admission, and hospitalization. According to the study, DM is associated with 2–3 times more risk of severe illness than non-diabetics; hence, the incidence of severe COVID-19 is higher in DM patients. Furthermore, the mortality rate in COVID-19 patients is much higher who had DM; estimations show that the risk of death is up to twice as high in COVID-19 patients as compared to the other population.^99^ In the case of COVID-19, the combination of insufficient immune responses, persistent inflammation, and other metabolic abnormalities associated with DM makes these people more vulnerable to severe and catastrophic consequences.^26^ The summary of impact of hyperglycemia on immune response against SARS-CoV-2 and its outcomes is summarized in Table 3.

**Table 3:** Comparison of immune responses and outcome in COVID-19 disease between diabetic and non-diabetic population.

No.	Immune features	Immune response against SARS-CoV-2 in non-DM individuals.	DM modified response against SARS-CoV-2.
1.	Basal immune state	Homeostatic innate immunity, lower baseline inflammatory markers.	Chronic low-grade inflammation (↑IL-6, TNF-α, CRP) predisposes to hyperinflammatory responses.
2.	Early innate sensing/Type I & III IFN	Rapid PRR sensing → timely type I/III IFN induction in many mild cases that helps control early viral replication.	Blunted/delayed type I IFN responses. Higher early viral replication and dysregulated downstream responses.
3.	Neutrophil numbers & function/NETs	Neutrophils recruited but regulated; NETs formed as needed.	Higher neutrophil activation and excessive NETosis. Contributes to lung damage and worse outcomes.
4.	Monocytes/Macrophages	Balanced macrophage activation and resolution in mild disease.	Skewing to pro-inflammatory phenotype (↑IL-6, IL-1β), impaired resolution — fuels cytokine storm risk.
5.	NK cells	Early NK cytotoxic responses help limit infected cells.	Reduced NK cytotoxicity and/or dysfunction in metabolic disease, reducing early viral clearance.
6.	Antigen presentation (APC function)	Effective antigen presentation promotes adaptive priming.	Impaired APC function and antigen presentation, delaying adaptive priming.
7.	CD4⁺ T-cell responses (helper)	Robust Th1 skewing in effective antiviral responses; supports B cells.	Impaired or delayed CD4 help; more T-cell activation/exhaustion markers in severe disease.
8.	CD8⁺ T-cell responses (cytotoxic)	Effective cytotoxic responses clear infected cells; correlate with milder disease.	Often lower or dysfunctional CD8 responses (lymphopenia and exhaustion in severe cases).
9.	B cells & antibody production	Neutralizing antibodies develop after infection/vaccination with generally good durability in healthy people.	Lower peak antibody titers or faster waning and reduced B-cell function after infection/vaccination in T2DM (especially with poor glycemic control).
10.	Vaccine response (magnitude/durability)	High seroconversion and T-cell responses with most vaccines; boosters improve breadth.	Reduced magnitude/durability (especially if poorly controlled). Resulting in higher breakthrough infection rates.
11.	Inflammatory markers during COVID-19	Mild/moderate elevations (IL-6, CRP) in non-severe cases.	Markedly higher IL-6, ferritin, CRP in severe disease.
12.	Clinal consequences	Lower baseline risk of severe COVID-19.	Higher risk of severe disease, ICU admission, thrombosis, and mortality; more frequent complications (e.g., ARDS, organ failure).

*Abbreviations*: DM, diabetes mellitus; IL, interleukin; TNF, tumor necrosis factor; CRP, C-reactive protein; IFN, interferons; PRR, pattern recognition receptors; NET, neutrophil extracellular trap; NK, natural killer; APC, antigen presenting cell; T2DM, type 2 DM.

### 4.4 DM and long COVID

DM, particularly T2DM, plays a complex role in long COVID, influencing both susceptibility and disease severity. DM and COVID-19 form a bidirectional relationship in which pre-existing DM predisposes to severe acute COVID-19, while SARS-CoV-2 infection may exacerbate glucose intolerance or precipitate new-onset DM.^100^ Mechanistically, chronic inflammation, ACE2 dysregulation, and β-cell dysfunction are key mediators in this interplay.^101^

Emerging cohort and registry data indicate that individuals with DM are more likely to experience persistent post-COVID symptoms including fatigue, dyspnea, headaches, and sleep disturbances for longer durations than non-diabetics.^102^ In India, diabetics had 2.3-fold increased odds of cardio-respiratory and 2.6-fold increased odds of neurological long COVID symptoms; hospital studies report up to 96% higher risk of ongoing symptoms at six months.^103^ A UK cohort similarly tracked long COVID diagnoses among thousands of people with both T1DM and T2DM.^104^ Moreover, a propensity-matched international registry analysis found that while readmission and reinfection rates post-COVID were similar between diabetics and non-diabetics, long-term mortality was higher in diabetics.^105^

Collectively, current evidence supports that DM exacerbates the risk and burden of long COVID through intertwined metabolic, inflammatory, and immune mechanisms. Clinical management strategies should emphasize tight glycemic control, rehabilitation, physical activity, and nutritional optimization to mitigate long-term sequelae.

## 5 DM and HIV/AIDS

### 5.1 Immune dysregulation in HIV

Infection by the HIV leads to persistently decreased levels of CD4 T cells, increased levels of CD8 T cells, and the inversion of CD4/CD8 ratio.^106^ CD4^+^ T-cell depletion is the hallmark of HIV infection. Furthermore, there is a substantial decline in proliferative capacity along with increased apoptosis of CD4^+^ T cells, increased expression of inhibitory molecules like cytotoxic T-lymphocyte-associated protein 4 (CTLA-4) and programmed cell death protein 1 (PD-1).^107^ Decreasing CD4^+^ T-cell counts in HIV infection leads to switch from Th1 to Th2 cytokine response, resulting in progressive decrease of IFN-γ levels and cytotoxic T cell functions and successive increase in IL-4 and IL-5.^107^ HIV infection is defined by impaired functioning of T lymphocytes, the underlying mechanism of which remains unclear.^108^ CD8 T-cell expansion being involved in the underlying mechanism of HIV development is present in all CD8 T cells, not only in those HIV specific. Several variables contribute to the CD8^+^ T cell proliferation particularly, increased levels of γ-common chain family cytokines, including IL-7 and IL-15, are important to consider. Moreover, IFN-1α, IFN-β and IL-12 have been found to be associated with CD8^+^ T cell expansion. Furthermore, during HIV infection, the over-activation of CD8^+^ T cells are associated with the activation of multiple immune cells which result in an inflammatory burden and contribute to non-AIDS-associated morbidity and mortality.^106^

Under physiological conditions, Th1/Th2 balance plays a significant role in maintaining peripheral tolerance and preventing excessive immune activation. This functionality is lost during HIV infection and leads to functional exhaustion HIV specific CD8^+^ T cells through PD-1/PD-L1 dysfunction.^109^

### 5.2 Prevalence of DM in HIV patients

WHO reports that about 39.9 million people around the globe were living with HIV by the end of 2023,^110^ making it a significant global health challenge. Literature shows that people living with HIV/AIDS are more susceptible to DM as compared to other populations. While around 537 million adults globally are affected by DM, the pathophysiology of HIV infection and DM comorbidity is not yet fully understood.^111^ It is thought that metabolic changes contributing to DM may arise from the chronic inflammation associated with HIV, as well as common risk factors like an unhealthy lifestyle, genetic predisposition, and aging.^111^ However, certain HIV-specific risk factors also appear to increase the prevalence of DM in people living with HIV. These include prolonged exposure to ART, particularly to “metabolically unfriendly” medications such as first-generation protease inhibitors and nucleoside analogues, as well as factors like disturbed lipid metabolism and persistent inflammation.^112^

Literature highlights that DM significantly affects people living with HIV, with an estimated comorbid frequency of up to 15% and an incidence of 10 per 1,000 person-years.^112^ Additionally, DM prevalence is projected to reach 9.4% in the USA, with even higher rate i.e. 15%—in the UK, North America, and Australia.^112^ A Brazilian cross-sectional study found that 7.14% of patients with HIV/AIDS also had DM.^111^ Another study in France suggested that an uncontrolled plasma viral load could increase the risk of developing DM, with evidence indicating a possible link between the route of HIV transmission and DM risk.^112^ Understanding the scope of this issue and implementing appropriate measures is essential to reduce DM complications, lower mortality rates, improve quality of life, and avoid potential negative impacts on HIV treatment outcomes.^113^

### 5.3 Impact of DM on the outcome of HIV

HIV targets CD^+^ T cells by binding molecules on the surface of Th cells, leading to replication of virus within these cells and their subsequent destruction. This results in a gradual decline in CD4^+^ T-cell counts. The “hyperimmune activation hypothesis” is another model explaining pathogenesis, suggesting that HIV infection triggers excessive cell division among CD4^+^ and CD8^+^ T-cells, NK cells, and B cells, accompanied by upregulation of activation markers. Chronic immune activation in HIV is characterized by elevated levels of circulating pro-inflammatory cytokines, chemokines, plasma proteins in early phase of infection. During acute phase, studies have also shown elevated anti-inflammatory cytokines such as IL-10, and IFN-inducible protein-10 (IP-10) which may be predicted as marker of early disease expansion.^114^

In the 1980s, the average life expectancy for individuals diagnosed with HIV/AIDS was approximately one year. However, advancements in medical care, particularly the early initiation of highly active antiretroviral therapy (HAART), have substantially improved survival rates, reduced mortality and extended the lifespan of individuals with HIV. Despite these benefits, HAART is associated with side effects including as insulin resistance, lipodystrophy, and dyslipidemia.^115^ Several studies have reported higher frequency of DM associated with HAART therapy. The growing concern about DM among individuals living with HIV aligns with its emergence as a major public health issue, particularly in countries with high HIV prevalence. In low- and middle-income countries where HIV rates are high, the burden of DM comorbidity is expected to rise, driven by an increasing incidence of DM.^116^

**Fig. 3:**
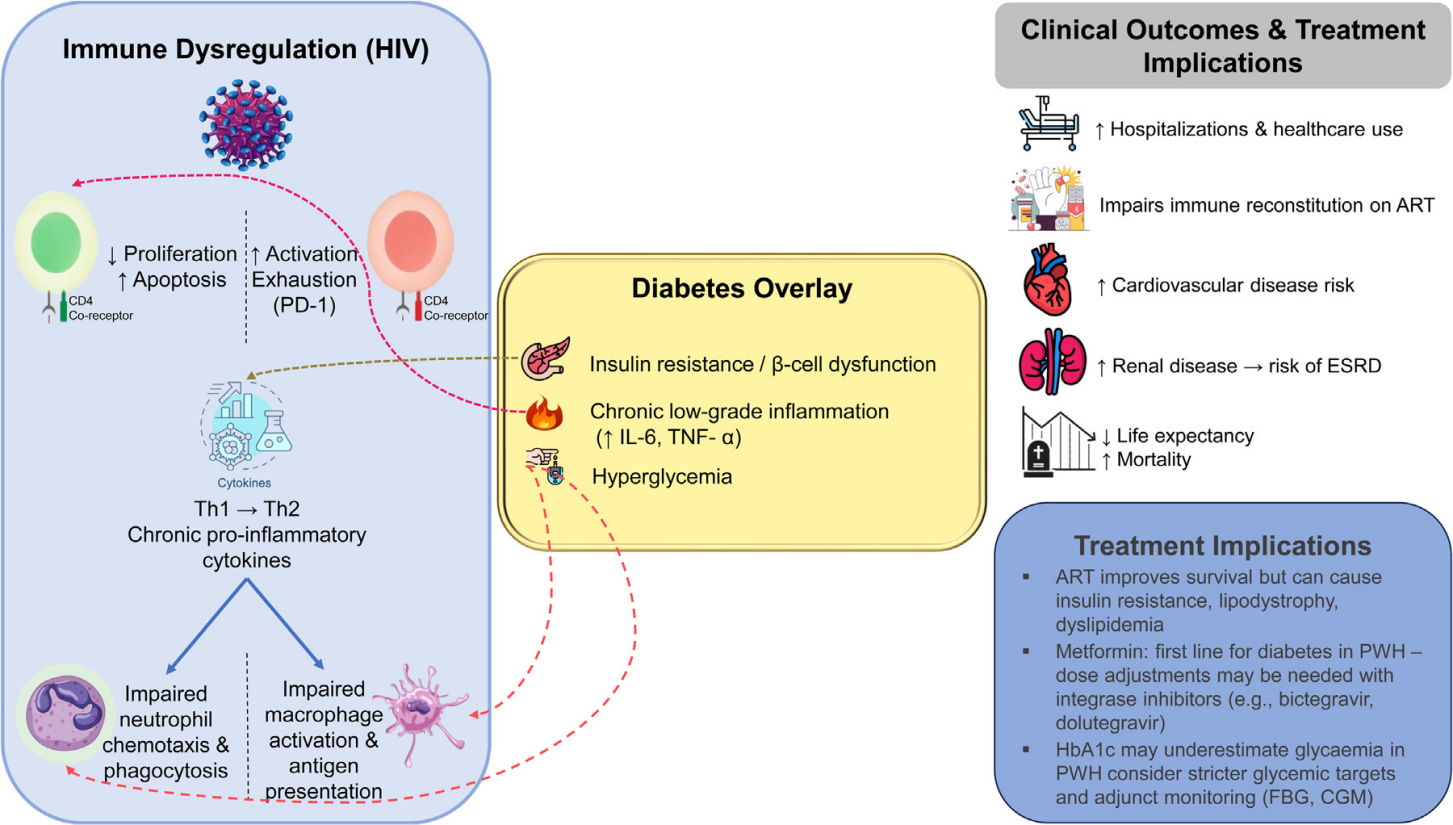
Clinical outcomes and treatment implication in HIV infected individuals with DM. *Abbreviations*: PD-1, programmed death receptor 1; Th, T-helper; IL, interleukin; TNF, tumor necrosis factor; DM, diabetes mellitus; ART, antiretroviral therapy; CVD, Cardiovascular disease; PWH, people with HIV; FBG, fasting blood glucose; CGM, continuous glucose monitoring; ESRD, end-stage renal disease.

While mortality related to HIV has significantly improved, individuals with coexisting DM face increased health challenges.^117^ HIV affects every organ system, from infancy to old age.^118^ The combined effects of HIV infection, ART toxicity, polypharmacy, social isolation, stigma, and other less recognized risk factors contribute to the complex health profiles of HIV-infected adults. Chronic conditions such as DM severely impact quality of life. DM in HIV patients is associated with increased risks of hospitalization, unfavorable renal and cardiovascular outcomes, and progression to end-stage renal disease, resulting in a reduced life expectancy and increased healthcare expenses.^119^ Chronic comorbidities in HIV represent a significant public health concern, as they are linked to greater healthcare needs, reduced overall quality of life, and increased mortality.^120^ The summary of impact of hyperglycemia on HIV/AIDS is shown in Fig. 3.

### 5.4 Impact of DM on HIV treatment

Due to limited studies on DM treatment in people with HIV (PWH), most recommendations are adapted from general population research. Metformin is recommended as a first-line therapy for PWH with DM, but dose adjustments may be needed when used with antiretroviral drugs like bictegravir and dolutegravir. Sulfonylureas should be used cautiously due to their potential to cause weight gain, especially since ART itself is a risk factor for weight gain. Additionally, pioglitazone has shown potential benefits in managing HIV-associated lipodystrophy syndrome, a condition characterized by abnormal fat redistribution and associated with insulin resistance and cardiovascular risk.^121^

The increasing evidence of the coexistence of T2DM among people living with HIV/AIDS, often leads to the need for long-term combination ART.^122^ In PWH, hemoglobin A1c (HbA1c) may underestimate actual blood sugar levels, necessitating stricter glycemic targets. Achieving these targets can help reduce complications such as macrovascular issues (heart disease, stroke, and peripheral vascular disease), although evidence for this benefit is mixed. It also significantly lowers the risk of microvascular complications like retinopathy, neuropathy, and nephropathy. However, intensive glucose control carries risks, including severe hypoglycemia and increased mortality in some cases. Clinicians must carefully balance the benefits of stricter glycemic control with these potential risks to ensure safe and effective treatment.^123^

## 6 Impact of DM on vaccine efficacy

DM, through its effects on innate and adaptive immunity, can modulate the outcomes of vaccination. While vaccine responses may be somewhat attenuated, clinical protection remains strong, especially with booster strategies and modern vaccine platforms.^124^^,^^125^ Overall, DM negatively influences vaccine immunogenicity, especially when glycemic control is poor. For TB, COVID-19, and future HIV vaccines, optimizing metabolic control is crucial to maximize vaccine effectiveness.^126^

### 6.1 Impact of DM on TB vaccination

The Bacillus Calmette-Guérin (BCG) vaccine provides variable protection against pulmonary TB, and data on its immunogenicity in DM are limited. However, there is evidence that DM, especially T2DM, compromises protective immunity.

Animal models and human studies suggest that hyperglycemia impairs macrophage function and IFN-γ responses, both of which are critical for BCG-induced immunity.^127^ Although no conclusive evidence demonstrates that BCG is less effective in diabetic patients, there is growing interest in whether DM-related immune dysregulation may partly explain reduced TB vaccine efficacy in high-burden populations. Experimental studies suggest reduced delayed-type hypersensitivity and impaired mycobacterial clearance in diabetic hosts after BCG vaccination.^128^ However, ongoing studies of new TB vaccines (M72/AS01E, Bill & Melinda Gates Medical Research Institute, USA) are increasingly considering DM as a comorbidity that may affect outcomes.^129^

### 6.2 Impact of DM on COVID-19 vaccination

COVID-19 vaccines have proven highly effective in reducing severe disease and mortality in people with DM. Several studies show that people with DM (both T1DM and T2DM) mount adequate but sometimes quantitatively reduced antibody responses to SARS-CoV-2 vaccines.^130^ Poor glycemic control correlates with lower neutralizing antibody titers and T-cell responses.^131^ Importantly, booster doses restore immune protection to comparable levels. Clinical data consistently show that vaccination reduces hospitalization and death from COVID-19 in DM, making it a cornerstone of preventive care.^132^ Underscoring the importance of vaccination in preventing both acute infection and potential autoimmune sequelae,^125^ maintaining good glycemic control enhances immune responses to COVID-19 vaccines.

### 6.3 DM and HIV vaccination

There is currently no licensed HIV vaccine, but multiple candidates (vector-based, mRNA, protein subunit) are under trial.^133^ People with DM may represent a vulnerable subgroup for HIV vaccine development because immune dysregulation in them could blunt vaccine-induced cellular immunity.^125^ Lessons from influenza and hepatitis B vaccines show that DM is associated with reduced vaccine seroconversion,^134^ suggesting that HIV vaccine efficacy might be lower in this population. As HIV vaccine trials progress, consideration of DM as a modifying factor for immunogenicity will be important for future strategies.

### 6.4 Broader implications for vaccines and preventive strategies

Vaccination in DM is not only a matter of individual protection but also of reducing community transmission of high-burden infections. Optimizing glycemic control before and after vaccination may enhance immune responses.^135^ Furthermore, patients with DM should be prioritized for vaccination campaigns against TB, COVID-19, and HIV (when available) given their elevated infection risk. Clinical guidelines emphasize routine immunizations in DM as part of comprehensive preventive care.

## 7 Conclusion and way forward

We conclude that immune modulation in DM has profound inverse relation with outcome of endemic infectious diseases including TB, COVID-19 and HIV/AIDS. Chronic hyperglycemia alters adaptive and innate immune responses, leading to impaired pathogen clearance, dysregulated release of cytokines, and increased susceptibility to severe, persistent infections. This immune dysregulation impacts the treatment and outcome of infectious diseases. It also impacts the outcome of vaccination in these individuals. The degree of immunologic dysfunction is closely associated with poor glycemic control, emphasizing the need for strict metabolic regulation. To reduce these risks and enhance clinical outcomes, it is necessary to develop integrated management strategies that combine optimal glycemic control with specific immunomodulatory medications, early infection screening, and immunization programs. Further research is essential to understand precise immunopathological mechanisms and identify biomarkers for early risk stratification in diabetic populations.

## CRediT authorship contribution statement

**Uzair Abbas:** Writing – review & editing, Writing – original draft, Supervision, Project administration, Methodology, Conceptualization. **Harendra Kumar:** Writing – review & editing, Writing – original draft, Conceptualization. **Niaz Hussain:** Writing – review & editing, Writing – original draft, Project administration, Data curation. **Ishfaque Ahmed:** Writing – review & editing, Writing – original draft, Resources, Project administration, Conceptualization. **Rabeel Nawaz Laghari:** Writing – review & editing, Writing – original draft, Supervision. **Misha Tanveer:** Writing – review & editing, Writing – original draft, Data curation. **Mohammad Hadif:** Writing – review & editing, Writing – original draft, Validation. **Kashaf Fatima:** Writing – review & editing, Writing – original draft, Project administration. **Muhib Ullah Khalid:** Writing – review & editing, Writing – original draft, Data curation. **Khadija Anwar:** Writing – review & editing, Writing – original draft, Validation. **Mahtab Khan:** Writing – review & editing, Resources, Data curation.

## Informed consent

Not applicable.

## Organ donation

Not applicable.

## Ethical statement

Not applicable.

## Data availability statement

Not applicable.

## Animal treatment

Not applicable.

## Generative AI

Not applicable.

## Funding

None.

## Declaration of competing interest

The authors declare that they have no known competing financial interests or personal relationships that could have appeared to influence the work reported in this paper.
